# GANT61 Reduces Hedgehog Molecule (GLI1) Expression and Promotes Apoptosis in Metastatic Oral Squamous Cell Carcinoma Cells

**DOI:** 10.3390/ijms21176076

**Published:** 2020-08-24

**Authors:** Taís Bacelar Sacramento de Araújo, Leonardo de Oliveira Siquara da Rocha, Manuela Torres Andion Vidal, Paulo Lucas Cerqueira Coelho, Mitermayer Galvão dos Reis, Bruno Solano de Freitas Souza, Milena Botelho Pereira Soares, Thiago Almeida Pereira, Ricardo Della Coletta, Daniel Pereira Bezerra, Rosane Borges Dias, Clarissa Araújo Gurgel Rocha

**Affiliations:** 1 Gonçalo Moniz Institute, Oswaldo Cruz Foundation (IGM-FIOCRUZ/BA), Salvador 40296-710, Bahia, Brazil; taisbsa@gmail.com (T.B.S.d.A.); siquaradarocha@gmail.com (L.d.O.S.d.R.); manuela.andion@gmail.com (M.T.A.V.); paulocoelhoba@gmail.com (P.L.C.C.); miter@bahia.fiocruz.br (M.G.d.R.); brunosolanosouza@gmail.com (B.S.d.F.S.); milenabpsoares@gmail.com (M.B.P.S.); danielpbezerra@gmail.com (D.P.B.); rosanebd@gmail.com (R.B.D.); 2 Department of Propedeutics, Faculty of Dentistry, Federal University of Bahia, Salvador 40110-909, Bahia, Brazil; 3 Department of Pathology, School of Medicine, Federal University of Bahia (UFBA), Salvador 40110-909, Bahia, Brazil; 4 D’Or Institute for Research and Education (IDOR), Sao Rafael Hospital, Salvador 41253-190, BA, Brazil; 5 Institute for Stem Cell Biology and Regenerative Medicine, Stanford University School of Medicine, Stanford, CA 94305, USA; dealmeida.thiago@gmail.com; 6 Department of Oral Diagnosis, School of Dentistry, University of Campinas, Piracicaba 13414-903, Sao Paulo, Brazil; coletta@unicamp.br

**Keywords:** oral neoplasms, Hedgehog signaling pathway, Hedgehog signaling inhibitors, targeted cancer therapy

## Abstract

Due to its importance in the pathogenesis of oral squamous cell carcinoma (OSCC), the Hedgehog (HH) pathway is considered a potential therapeutic target. We investigated the effects of GANT61, a GLI inhibitor, on HH gene expression, as well as on metastatic OSCC cell proliferation and death. Following culture in DMEM medium, cytotoxicity of GANT61 against different tumor and non-tumor cell types was assessed by alamarBlue assays. Cytotoxicity analysis revealed that the metastatic HSC3 cell line was the most sensitive (IC_50_: 36 µM) to the tested compound. The compound’s effects on the expression of HH pathways components were analyzed by qPCR and Western blot; cell viability was analyzed by trypan blue assay and flow cytometry were used to investigate cell cycle phase, morphology, and death patterns in HSC3 cells. A significant reduction in mRNA levels of the GLI1 transcription factor was found after 12 h of treatment withGANT61. Protein expression levels of other HH pathway components (PTCH1, SHH, and Gli1) and HSC3 cell viability also decreased after 24 h of treatment. Cell cycle analysis and death pattern evaluations revealed significantly increased nuclear fragmentation in sub-G1 phase, as well as cell death due to apoptosis. In conclusion, the significantly reduced GLI1 gene expression seen in response to the GLI inhibitor indicates diminished downstream activation in HH pathway components. GANT61 significantly reduced cell viability in the metastatic cell line of OSCC and promoted a significant increase in nuclear fragmentation and cell death by apoptosis.

## 1. Introduction

Cancer remains the second leading cause of mortality worldwide, accounting for one in six, or 9.6 million, deaths reported in 2018 [1]. With respect to malignant neoplasms of the oral cavity, oral squamous cell carcinoma (OSCC) is the most common histological subtype, representing more than 90% of diagnosed oral cancer cases [2,3,4].

Although clinical outcomes for patients with locally advanced OSCC have improved significantly over the years, due to multimodal cancer therapy, this is not the case for patients who present recurrence and/or distant metastases [5]. The high morbidity and mortality of OSCC occurs mainly due to late diagnosis, contributing to a high rate of metastatic cases. Moreover, limited knowledge concerning this tumor’s biology has led to a consequent lack of potential target molecules that may direct more effective therapeutic strategies [6,7]. Thus, achieving a fuller understanding of the biological processes underlying treatment effectiveness and the development of metastasis is essential to the success of intervention strategies.

Of the new therapeutic approaches currently under investigation, with many endeavors to inhibit the signaling pathways involved in the initial stages of tumor development, notable emphasis placed on the Hedgehog (HH) embryonic signaling pathway, which becomes reactivated in OSCC and has been demonstrated to contribute to progression in this tumor type [5,8,9,10].

HH pathway activation can occur through both classical (or canonical) and non-canonical signaling. Activation via the canonical route involves three main ligands: Sonic Hedgehog (SHH), Desert Hedgehog (DHH), and Indian Hedgehog (IHH), in addition to the Patched1 (PTCH1) and Smoothened (SMO) receptors [11,12]. Interaction between HH ligands and the PTCH1 receptor leads to the release of the SMO protein, which accumulates in the primary cilium, triggering intracellular cascades that culminate in the activation of the *Glioma*-associated oncogene (GLI) family transcription factors (GLI1, GLI2, and GLI3), which then translocate to the nucleus. The GLI1 protein mainly acts as a transcriptional activator, participating in the activation of genes favoring the aberrant proliferation (cyclin D1 and cyclin D2, N-myc), survival (BCL2), and maintenance of the tumor stem cell population, while GLI2 and GLI3 exhibit activating and repressive functions. In contrast, the “non-canonical” activation of the HH pathway occurs in a ligand-independent manner and can be triggered through the inactivation of PTCH1 (Type I) and/or SMO (Type II), or via direct stimulation of transcriptional GLI factors via other signaling pathways, as a “bypass”, occurring independently of the signaling mediated by the PTCH1/SMO complex [11].

To date, most upstream investigations of HH pathway inhibition targets have focused on FDA-approved SMO inhibitors, especially cyclopamine, itraconazole, and vismodegib [13,14]. However, the pharmacokinetic properties of these drugs, such as high cytotoxicity, low solubility, and chemical instability against OSCC and other tumor cells, have limited therapeutic applications. Furthermore, resistance to SMO inhibitors has already been observed in patients with basal cell carcinoma (BCC) [15]. It has been reported that the downstream inhibition of the HH pathway through the targeting of GLI1 can alternatively attenuate the expression of genes involved in the biological behavior of cancer, as well as interfere with other signaling cascades involved in tumor progression, such as *MAPK*, *PI3K*, and *TGFβ* [11,16]. Thus, the downstream inhibition of HH pathway regulators by GLI transcription factor antagonists, especially the efficacious activity demonstrated by the experimental agent GANT61, may present a promising strategic alternative to SMO inhibitors [17].

GANT61 is a synthetic compound derived from hexahydropyrimidine, notable for its efficient binding to GLI transcription factors, as well as to the GLI-DNA complex [18]. On the nuclear level, GANT61 binds to GLI, in close proximity to, but independent from, the DNA binding region. Studies have shown that GANT61 significantly decreases the transcriptional production and gene expression of *GLI1*, *PTCH1*, and other HH pathway target genes, as evidenced by inventoried GLI assays in a range of cancer cell types [17,18,19,20]. Yan et al. (2011) presented preliminary results concerning this issue in two non-metastatic OSCC cell lines, CAL-27 and SCC-5 [21]. To the best of our knowledge, no studies to date have specifically focused on the therapeutic potential of this GLI inhibitor in the context of metastatic OSCC.

Thus, the purpose of this work was to evaluate the antitumoral activity of GANT61, a downstream inhibitor of the HH pathway, on the gene and protein expression of HH pathway components (PTCH1, GLI1, GLI2, and GLI3), as well as tumor cell proliferation and death in a metastatic OSCC cell line.

## 2. Results

### 2.1. HSC3 Cells Show Nuclear GLI1 and Cytoplasmic Expression of SHH, PTCH1, SMO, and GLI1

**Figure 1:**
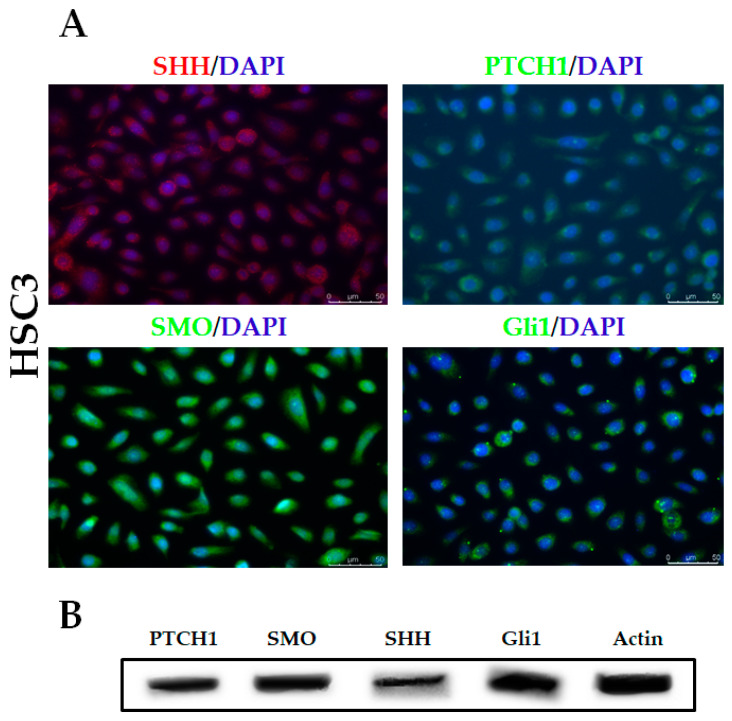
Evaluation of HH protein (SHH, PTCH, SMO, and Gli1) expression in HSC3 cells by immunofluorescence (**A**) and Western blot (**B**). Actin was used as an endogenous control for the Western blot assay.

Our results demonstrate the cytoplasmic presence of all essential components of the HH pathway (SHH, PTCH1, SMO, and GLI1) in HSC3 cells, as well as the nuclear expression of the GLI1 protein (Figure 1A). In addition, Western blotting confirmed the presence of all of these Hedgehog proteins in HSC3 cells (Figure 1B).

### 2.2. GANT61 Demonstrated Significant Cytotoxicity against a Panel of Human Tumor and Non-Tumor Cell Lines

The cytotoxic activity of the GLI1 inhibitor was evaluated after 72 h of incubation in different human tumor cell lines, using an alamarBlue assay. For comparative analysis, cytotoxic activity was also evaluated in non-tumor cells (NOF, HaCaT, and PBMC) (Table 1). Cytotoxic concentrations of GANT61 were determined by using IC_50_ values ranging from 36 to 110.6 µM for the HSC3 and SCC4 lines, respectively. Moreover, 5-fluorouracil (5-FU) was used as a positive control, since this drug is commonly used for the treatment of advanced OSCC in clinical settings. Cytotoxicity was also demonstrated in the positive control at IC_50_ values anging from 1.3 to 143.2 µM for the HepG2 and SCC9 lines, respectively, with a corresponding IC_50_ of 17 µM for the HSC3 line. Doxorubicin, a classical chemotherapeutic agent used as positive control in cytotoxicity assays, showed values ranging from 0.01 to 6.8 µM for the HepG2/HL60 and APC02 lines. Since HSC3 cells demonstrated greater sensitivity to GANT61 than the other OSCC cell lines evaluated, and the fact that this cell type exhibited higher gene expression of all GLI transcription factors (*GLI1*, *GLI2*, and *GLI3*) (Supplementary Figure S1), we accordingly selected this cell line for use in all following in vitro assays, which allowed us to better evaluate the effects of GANT61 on HH pathway component gene expression, as well as cell viability, cycle, and death patterns.

### 2.3. GANT61 Reduced mRNA Levels of GLI1 Transcription Factor

**Figure 2:**
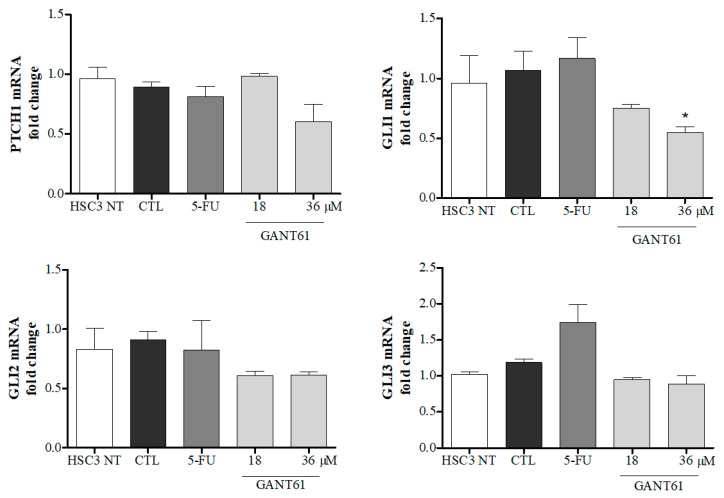
Gene expression of HH pathway components PTCH1, GLI1, GLI2, and GLI3 after 12 h of treatment with GANT61 (18 and 36 µM). Negative control was treated with DMSO (0.2%), used to solubilize and dilute tested compounds; 5-FU (17 µM) was used as positive control. Relative quantification (RQ) values used in each sample were normalized by using the B2M reference gene and calibrated according to RQ values obtained for the HSC3 non-treated cell group (HSC3 NT); qPCR reactions were performed in GANT61-treated and non-treated cells. * *p* < 0.05 when compared to the negative control group by ANOVA (variance test) followed by the Student–Newman–Keuls test.

At the half-maximal inhibitory concentration of GANT61 (36 µM), a statistically significant reduction was observed in mRNA levels of the *GLI1* transcription factor in comparison to negative (DMSO 0.2%) and positive (5-FU) controls. Reductions in mRNA expression were detected in the other genes evaluated at both concentrations tested, with the exception of *PTCH1* at 18 µM, despite the lack of significant differences (Figure 2).

### 2.4. GANT61 Reduced the Expression of HH Pathway Proteins (PTCH1, SHH and Gli1)

**Figure 3:**
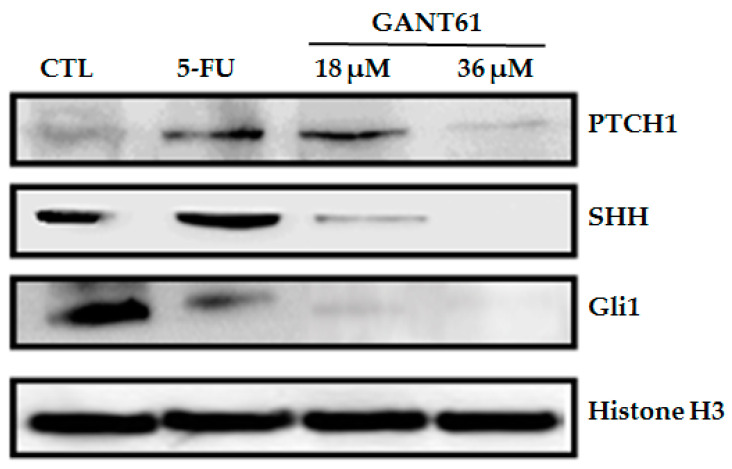
Effect of GANT61 on protein levels of selected HH components (PTCH1, SHH, and Gli1) as determined by Western blot after 24 h of treatment. The negative control (DMSO, 0.2%) was used to solubilize and dilute all tested compounds; 5-FU (17 µM) was used as a positive control; and Histone H3 was used as endogenous control.

After 24 h of GANT61 treatment at both tested concentrations (18 and 36 µM), reduced levels of PTCH1, SHH, and Gli1 protein expression were observed in HSC3 cells (Figure 3). The positive control (5-FU) was also shown to reduce Gli1 protein levels.

### 2.5. GANT61 Significantly Reduced the Viability of HSC3 Cells

**Figure 4:**
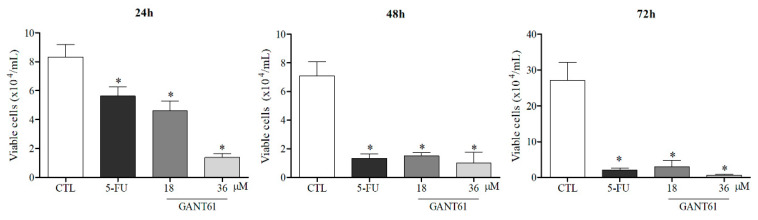
Effects of GANT61 treatment on HSC3 cell viability as determined by trypan blue exclusion assay after 24, 48, and 72 h of treatment. The negative control (DMSO, 0.2%) was used to solubilize and dilute all tested compounds; 5-FU (17 µM) was used as a positive control. Values correspond to the mean + SEM of three independent experiments performed in duplicate. * *p* < 0.05 when compared to the negative control group by ANOVA (variance test), followed by the Student–Newman–Keuls test.

Treatment with the compound (for 24, 48, and 72 h) at both evaluated concentrations (18 and 36 µM) was shown to significantly reduce the viability of HSC3 cells compared to the negative control (0.2% DMSO) (Figure 4). The 5-FU positive control also reduced the number of viable cells at all incubation times. No significant differences were found with regard to the number of non-viable cells between groups.

### 2.6. GANT61 Caused Cell Shrinkage and Nuclear Fragmentation in HSC3 Cells

**Figure 5:**
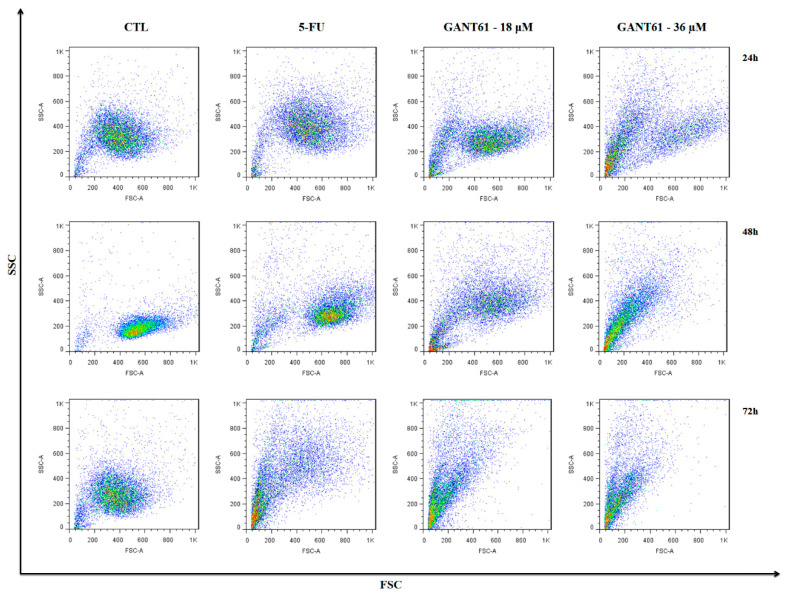
Dot plots representing light dispersion characteristics in HSC3 cells treated with GANT61. FSC (forward scatter) and SSC (side scatter), as determined by flow cytometry, were used to demonstrate relative size and granularity or internal cell complexity, respectively, after 24, 48, and 72 h of treatment. The negative control (DMSO, 0.2%) was used to solubilize and dilute the tested compounds; 5-fluorouracil (5-FU, 17 µM) was used as positive control. Data represent results from three independent experiments performed in duplicate. Cell debris was omitted from analyses; 10,000 events were analyzed per sample.

The treatment of HSC3 cells with GANT61 (18 and 36 µM) caused cell shrinkage, observed by a decrease in forward scatter (FSC) and nuclear fragmentation, as well as increased side scatter (SSC) (Figure 5). The 5-FU treatment group also exhibited changes consistent with apoptotic death. Flow cytometric readings indicate that morphologic effects on HSC3 cells were concentration- and time-dependent.

### 2.7. GANT61 Decreased HSC3 Cells in G0-G1 Phase and Increased Fragmentation of Internucleosomal DNA

**Figure 6:**
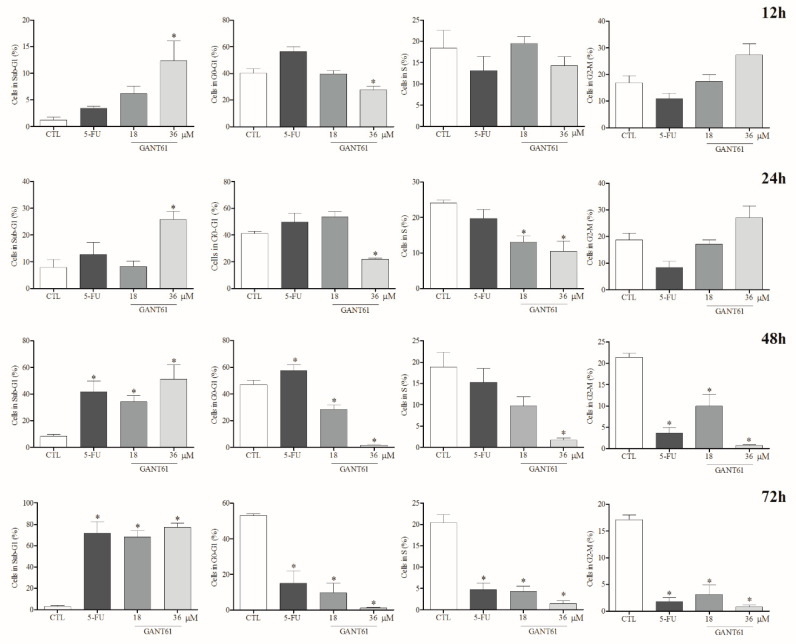
GANT61 effects on cell cycle and internucleosomal DNA fragmentation in HSC3 cells. Values correspond to the average + SEM of three independent experiments performed in duplicate. The negative control (DMSO, 0.2%) was used to solubilize and dilute the tested compounds; 5-FU was used as positive control. Cell debris was omitted from analyses; 10,000 events were analyzed per sample. * *p* < 0.05 when compared to the negative control group by ANOVA (variance test), followed by the Student–Newman–Keuls test.

Figure 6 depicts the percentages of HSC3 cells in each respective cycle and internucleosomal DNA fragmentation following GANT61 treatment. After 12 h of incubation with 36 µM of GANT61, a significant increase was observed in the percentage of HSC3 cells in the sub-G0/G1 phase, which represents an increase in nuclear DNA fragmentation, a strong indicator of apoptotic death. From this time point forward, a significant decrease in the number of cells in the G0-G1 phase treated with GANT61 (36 µM) was observed, suggesting the possibility of cell cycle arrest in this phase. At earlier time points (24 and 48 h), significantly higher numbers of cells were found in the sub-G0/G1 phase in the group treated with GANT61 (36 µM), in comparison to the negative control (DMSO 0.2%). In addition, at later time points (≥48 h), significantly lower numbers of cells in S and G_2_/M phases were seen in the positive control (5-FU) and GANT61 (18 and 36 µM) treatment groups.

### 2.8. GANT61 Induced Apoptosis in HSC3 Cells after 24 h of Treatment

**Figure 7:**
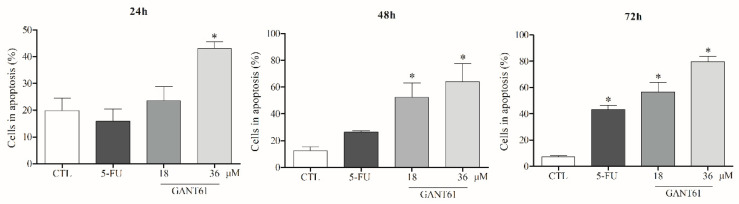
GANT61 effects on phosphatidylserine externalization in HSC3 cells determined by flow cytometry, using Annexin V-FITC. Values correspond to the average + SEM of three independent experiments performed in duplicate. The negative control (DMSO, 0.2%) was used to solubilize and dilute the tested compounds; 5-FU was used as positive control. Cell debris was omitted from analyses; 10,000 events were analyzed per sample. * *p* < 0.05 when compared to the negative control group by ANOVA (variance test) followed by the Student–Newman–Keuls test.

**Figure 8:**
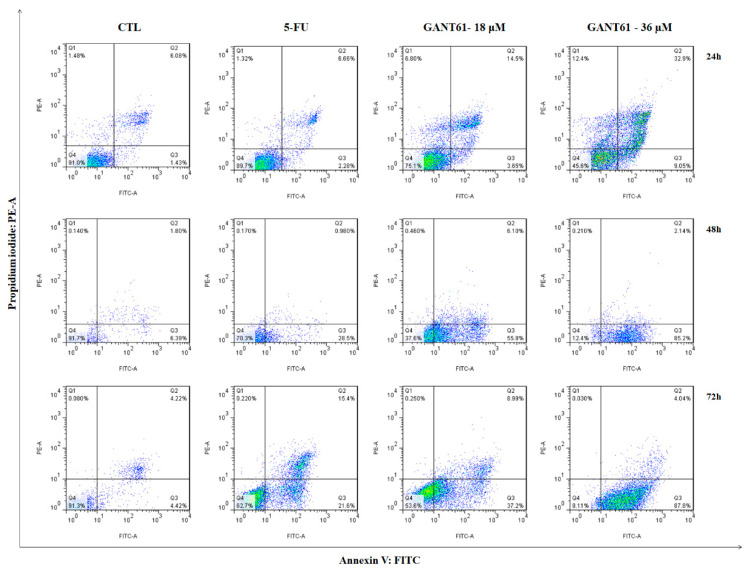
Representative flow cytometry dot plots show the percentage of cells in viable, early apoptotic, late apoptotic, and necrotic stages in HSC3 cells treated with GANT61 after 24, 48, and 72 h of treatment. The negative control (DMSO, 0.2%) was used to solubilize and dilute all tested compounds; 5-fluorouracil (5-FU, 17 µM) was used as positive control. Data represent results from three independent experiments performed in duplicate. Cell debris was omitted from analyses; 10,000 events were analyzed per sample.

The treatment of HSC3 cells with GANT61 at both concentrations was shown to considerably decrease cell viability. Our findings indicate significantly higher exposure of phosphatidylserine on HSC3 cell surfaces in the group treated with GANT61 (36 µM), at all times evaluated, compared to the negative control group DMSO (0.2%). At a concentration of 18 µM, treatment with GANT61 significantly increased cell apoptosis at 48 and 72 h of incubation. By contrast, significantly increased cell apoptosis was only evident in the positive control group (5-FU) after 72 h of incubation (Figure 7 and Figure 8).

## 3. Discussion

In the context of oral squamous cell carcinoma, disease severity has been positively correlated with the canonical activation of the Hedgehog pathway, occurring independently of PTCH1/SMO complex signaling. Non-canonical HH pathway activation occurs through activation by members of the *glioma* gene family (*GLI*), which are known to participate in the initiation, progression, and maintenance of tumor stem cells, as well as therapeutic resistance [11,15,16]. Thus, considering that downstream inhibition represents a more targeted approach in the blockade of the HH pathway, the present study aimed to evaluate the antitumoral activity of a GLI inhibitor (GANT61), as well as its effects on gene expression of HH pathway components and OSCC cell proliferation and death.

Previous results from our group demonstrated the reactivation of the HH pathway in OSCC [8]. Herein, HH pathway activation was determined through the detection of HH protein expression, especially nuclear GLI1, as well as other HH pathway components (SHH, PTCH1, SMO, and GLI1), providing evidence that HSC3 cells are capable of responding to exogenous signaling, culminating in the activation of this signaling cascade.

Initially, the present study employed a cytotoxicity assay in a panel of tumor and non-tumor cells, as well as on primary-oral-cancer-associated fibroblasts associated with oral cancer (CAF 1 and CAF2). Our results revealed that the highly metastatic OSCC line HSC3 presented greater sensitivity to GANT61, as compared to the other tumor cell lines tested. Accordingly, this line was selected for all subsequent in vitro studies.

The antitumor effects of GANT61 were reported in the SCC-25 and CAL27 OSCC cell lines, in comparison to an upstream HH inhibitor (Cyclopamine) [21]. While these authors tested different concentrations of GANT61 (2, 5, and 10 µg/mL, corresponding to 4.7, 11.6, and 23.3 on a µΜ scale), the method chosen to determine IC_50_ values was not clearly described. In the present assessment, with the exception of CAL27, all tested OSCC cell lines were found to be sensitive to GANT61, with the lowest IC_50_ found for HSC3. In contrast to this highly metastatic cell type, the cell lines investigated by Yan et al. (2011) exhibit different biological behavior [22,23]. For instance, CAL27 is non-metastatic and forms well-differentiated squamous cell carcinoma, while SCC-25 experiments in nude mice revealed the formation of well-organized differentiated cysts and encapsulated tumor masses [24,25,26].

The cytotoxic effects of GANT61 have been evaluated in different histological tumor types, such as neoplasms of the colon, cervix, pancreas, lung, pleural mesothelium, and biliary tract, as well as in neuroblastoma, myeloid leukemia, and rhabdomyosarcoma. The IC_50_ values calculated for this GLI inhibitor have ranged from 5 to 15 µΜ in most studied cell lines [18,19,20,27,28,29,30,31,32]. This study conducted in vitro testing, using the presently determined IC_50_ concentration (36 µΜ), as well as half of this amount (18 µΜ). At both analyzed concentrations, HSC3 cells exhibited reduced cell viability and increased nuclear fragmentation and apoptosis. It is worth mentioning that the pharmacological effects of GANT61 were found to be superior to those observed when using the positive control (5-FU), which is an adjuvant component of the only combined chemotherapy regimen (DCT (Docetaxel), Cisplatin, and 5-Fluorouracil) approved by the US Food and Drug Administration for patients with head and neck squamous cell carcinoma. However, this treatment has been shown to impact patient survival modestly, in addition to being associated with high systemic toxicity and death [33,34].

HH pathway gene (*PTCH1*, *GLI1*, *GLI2*, and *GLI3*) and protein (PTCH, SHH, and GLI1) expression were evaluated by qPCR and Western blot after 12 and 24 h of treatment with GANT61, respectively, using two concentrations (18 and 36 µM). Reduced levels of mRNA were detected in all evaluated genes, compared to the DMSO control group, with the exception of PTCH1 at a concentration of 18 µM. Notably, soon (12 h) after treatment with this GLI inhibitor at 36 µM, a statistically significant reduction in the mRNA levels of *GLI1* was observed in HSC3 cells. Protein levels of PTCH1, SHH, and GLI1 were also found to decrease substantially after 24 h of treatment. It has been clearly established that GLI1 gene and protein expression is considered the gold standard for determining HH pathway activation [35,36,37,38,39,40]. Herein, the absence of statistical significance with regard to GLI2 and GLI3 could be related to intrinsic limitations of in vitro assays, such as the number of replicates typically performed. Some authors have suggested that GANT61 acts selectively by inhibiting *GLI1*- and *GLI2*-mediated gene activation in different tumor cells [18,19,28,31,32,41]. On the other hand, the regulation of PTCH1 expression is known to be comparatively more complex and has been shown to involve feedback loops [40,42].

Our findings are similar to data published by Lim et al. (2015), who analyzed medulloblastoma cells treated with GANT61 for 24 h and reported significantly inhibited gene and protein expression of GLI1 and GLI2, as well as reduced mRNA levels of the oncogene *BCL2* at all tested concentrations (10, 20, and 40 µM). These authors suggested that negative regulation of *BCL2* may be responsible for the observed increase in cell death, following GLI suppression, as well as related DNA damage [41]. In addition, Yan et al. (2011) also described reduced GLI1 transcription activity in non-metastatic OSCC lines CAL27 and SCC-25, using a GLI-BS-Luciferase reporter assay, as well as a reduction in protein expression in some target genes of the HH pathway: *GLI1*, *GLI2*, and *Cyclin D1* [21].

In the different breast cancer cell lines assessed by Kurebayashi et al. (2017), including tumor stem cell lines, GANT61 was found to significantly decrease the levels of GLI1 and GLI2 expression, but not GLI3, in a dose-dependent manner [16]. Similar results were observed in other human cancer lines after treatment with GANT61, with inventoried GLI assays demonstrating a reduction in gene and protein expression of the target genes GLI1 and PTCH1, as well as reduced transcriptional production [18]. In pancreatic tumor stem cells, treatment with GANT61 (10 µM) inhibited the expression of factors *GLI1* and *GLI2*; *PTCH1* and *PTCH2*; and SMO. In addition, since SHH expression was not detected in treated cells, these authors suggested that GANT61 may regulate the characteristics of tumor stem cells by inhibiting components of the HH pathway [28].

The present tumor cell viability assays demonstrated GLI inhibition by GANT61 in a time- and concentration-dependent manner, as evidenced by significantly reduced numbers of viable HSC3 cells compared to negative controls after 24 h of treatment at both evaluated concentrations. A study by Kurebayashi et al. (2017) reported that, even at low concentrations (0.1 to 1 µΜ), GANT61 did not affect the growth of any of the tested breast-cancer cell lines positive for estrogen receptors, whether under estrogen deprivation or estrogen supplementation conditions. Moreover, high concentrations (5–20 µΜ) of GANT61 were shown to inhibit tumor growth in a concentration-dependent manner [16]. Another study that also used different breast cancer lines reported a significant reduction in cell viability in 7/8 cell lines examined, in response to treatment with GANT61 (5 to 20 μM, 48 and 72 h) versus DMSO, the negative control, confirming inhibition in a time- and concentration-dependent manner [43].

Similarly, Fu et al. (2013) used a trypan blue assay to evaluate the viability of pancreatic tumor stem cells treated with GANT61, both in 2D (monolayer) and 3D model cultures (spheroids in suspension), at concentrations of 1.0, 5.0, and 10 µM. These authors observed a significant reduction in viable cells in a time- and concentration-dependent manner (48 and 72 h), in addition to the induction of apoptosis [28]. Corroborating these findings, a study by Lin et al. (2016) evaluated different concentrations of GANT61 (10, 20, and 40 µM) with respect to medulloblastoma cell viability, reporting both a statistically significant reduction in populations of viable cells and a considerable increase in the number of cells undergoing apoptosis after 24 h of treatment [44].

The present study demonstrated a significant increase in nuclear fragmentation, a key feature of apoptosis, in sub-G1 phase after 12 h of treatment with the highest concentration of GANT61 (36 μM). Moreover, a statistically significant reduction in G1 and S phases was seen after 12 h of treatment, and in G2/M phases after 48 h. These results are similar to those reported by Lim et al. (2015), who evaluated the antiproliferative action of GANT61 (20 μM) in malignant mesothelioma cells. These authors observed a G1 phase halt after 24 h of treatment, compared to the negative control, in addition to cell accumulation in the sub-G1 fraction between 48–72 h, suggestive of death by apoptosis [41]. Further corroborating our results, Kurebayashi et al. (2017) also demonstrated that GANT61 increased the proportion of cells in sub-G1 phase, decreased cell accumulation in S phase, and delayed the G1-S cell cycle in all breast cancer cell lines evaluated in a concentration-dependent manner. Moreover, GANT61 also increased apoptosis in all tested cell lines when combined with an apoptotic-inhibition suppressor (survinin), as evidenced by annexin V/PI staining [16].

Our evaluation of the cell-death profile following treatment with GANT61 indicated apoptotic death occurring due to significant increases in cells with phosphatidylserine exposure after 24 h of treatment. Similarly, Lim et al. (2015) observed a parallel increase in apoptotic cell death in a time-and concentration-dependent manner in malignant mesothelioma cells treated with GANT61 [41]. It is notable that both concentrations of the GLI inhibitor tested herein gave rise to apoptotic activity superior to the positive control, 5-FU, which only produced a statistically significant increase in apoptosis after 72 h of treatment. With respect to the morphology of the HSC3 cells treated with GANT61, flow cytometry readings indicated considerable cell shrinkage and increased cell granularity in a time- and concentration-dependent manner, which is consistent with our findings of increased internucleosomal fragmentation and reduced cell viability.

The present results demonstrate the therapeutic potential of GLI1 inhibition, using GANT61 as a strategy to treat OSCC in 2D in vitro assays, with greater sensitivity exhibited by HSC3 metastatic cells. In addition, the observed cytotoxicity of GANT61 in CAFs and NOF raises relevant scientific questions regarding the possibility of employing the HH pathway as a common target in malignant and stromal cells.

**Figure 9:**
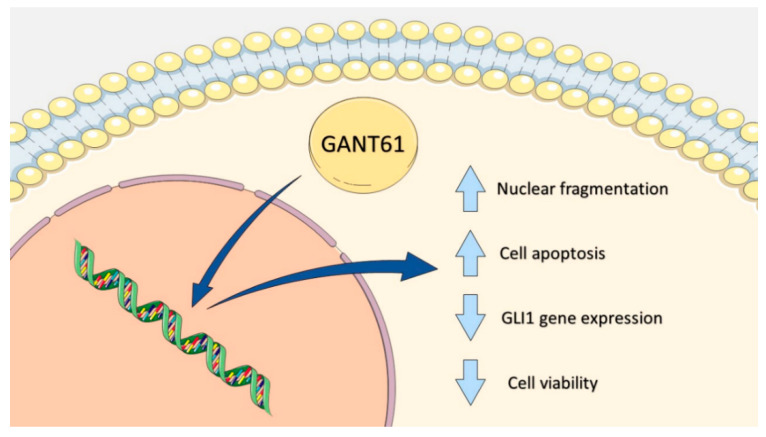
Summary of therapeutic effects of GANT61 treatment in metastatic HSC3 cells. Note: image created by the authors. Graphic sources available at: https://smart.servier.com/, accessed on June 13, 2020. A service to medicine provided by Les Laboratoires Servier (www.servier.com). License: https://creativecommons.org/licenses/by/3.0/.

Pharmacological inhibition by GANT61 was found to reduce GLI1 gene and protein expression in highly metastatic HSC3 cells, indicating decreased signaling cascade activity after 12 and 24 h of treatment, respectively. Moreover, GANT61 was also shown to reduce tumor cell viability, alter cell morphology and induce apoptosis in the HSC3 cell line evaluated (Figure 9).

## 4. Materials and Methods

### 4.1. Cell Culture Acquisition and Maintenance

For cytotoxicity assessments, this study primarily focused on the highly metastatic HSC3 OSCC cell line (JCRB Cell Bank, Osaka, Japan) and employed other histological tumor cell types and non-tumor cells (Supplementary Table S1). HSC3 cells were placed in flasks (75 cm^3^, 250 mL volume) containing DMEM medium (Gibco, Lif Technologies, Gaithesburg, MD, USA) supplemented with 10% fetal bovine serum (FBS, Gibco, Life Technologies, Gaithesburg, MD, USA) 1% penicillin, 1% streptomycin (Gibco, Life Technologies, Gaithesburg, MD, USA), and 0.8% hydrocortisone (Sigma-Aldrich, St. Louis, MO, USA). Cells were cultured and kept in incubators, under an atmosphere of 5% CO2 at 37 °C. Trypsinization was used to dissociate cells when cell growth reached the necessary confluence, i.e., 70 to 80% of the total culture flask volume. Cell cultures were tested periodically for mycoplasma contamination and evaluated by using a luminometer, according to the MycoAlert^TM^ PLUS protocol (Lonza, Walkersville Inc., Walkersville, MD, USA, 2012).

#### PBMCs from Healthy Volunteers

Human peripheral blood mononuclear cells (PBMC) lymphocytes and monocytes) were obtained from the peripheral blood of healthy, non-smoking volunteers aged 25–35 years. None of the volunteers had used any type of drug or medication for at least 15 days prior to collection. Blood collection (5 mL) was performed in heparinized vials by trained professionals at the Laboratory of Tissue Engineering and Immunopharmacology (LETI, IGM-FIOCRUZ, Salvador, Brazil), using sterile disposable syringes. PBMCs were isolated by a standard protocol described by Dias et al. (2018) [6]. The institutional review board of the Gonçalo Moniz Institution, Oswaldo Cruz Foundation (IGM-FIOCRUZ), in Salvador, Bahia, Brazil, approved all experimental protocols (No.031019/2013). All participants provided written informed consent prior to participation in the study.

### 4.2. Acquisition of Cancer-Associated Fibroblasts (CAF), CAF-Conditioned Medium, and Normal Fibroblasts

Two cancer-associated fibroblast populations (CAF1 and CAF2) were obtained as described by Sobral et al. (2011), from fragments of squamous cell carcinoma of the tongue that were removed from patients seen at the UNICAMP School of Dentistry in Piracicaba, São Paulo, Brazil [45]. 

Specimens were washed with PBS, fragmented, and placed in 25 cm^2^ cell culture flasks containing 1 mL of DMEM medium supplemented with 10% FBS and an antibiotic, and then maintained in an incubator, at 37 °C, under 5% CO_2_. Cell growth was monitored daily, and the medium was replaced every 2–3 days. When the cells reached a certain confluence, they were trypsinized, propagated, and characterized as fibroblasts or myofibroblasts by immunocytochemistry, flow cytometry, and Western blot analysis. Type I collagen production as assessed by ELISA was also used as a marker of myofibroblast activity. Both CAF1 and CAF2 primary populations were maintained for a maximum of 12 passages, with CAF phenotype maintenance evaluated through immunocytochemical staining for the α-SMA antibody after each two passages. To prepare the CAF-conditioned medium, fibroblasts were kept in culture until reaching 70–80% confluence. Culture flasks were then washed with saline, to remove FBS residue, and filled with serum-free DMEM medium for 72 h. The medium was collected, centrifuged for 10 min at 10,000 rpm, to remove any cellular debris, and stored in 1 mL aliquots, at a temperature of −80 °C.

For comparative analysis, primary cultures of normal oral fibroblasts (NOF) were isolated from fragments of healthy patient gingiva, using the same technique previously described for CAFs above, and then maintained in culture for up to 15 passages.

### 4.3. Cytotoxicity Assessments Using the AlamarBlue Assay and Drug Specifications

To assess the cytotoxicity of the GLI inhibitor GANT61 (Sigma-Aldrich, St. Louis, MO, USA) in tumor and non-tumor cell lines, an alamarBlue assay (Sigma-Aldrich Chemical Co, St. Louis, MO, USA) was performed after 72 h of exposure to the inhibitor, as described by Ahmed et al., 1994 [46]. Cells were distributed on 96-well plates at a density of 7 × 10^4^ cells/mL for adhered cells, and 3 × 10^5^ cells/mL for cells in suspension. All tested compounds were dissolved in DMSO, added to wells containing 100 µL of cells in complete medium, and then incubated for 72 h. Eight concentrations of GANT61 (0.19 to 50 µg/mL), Doxorubicin (Laboratory IMA S.A.I.C., Buenos Aires, Argentina), and 5-Fluorouracil (5-FU, Sigma-Aldrich, St. Louis, MO, USA), both used as positive controls at concentrations ranging from 0.03 to 5 µg/mL, were added to respective wells. Cells treated with the vehicle (0.5% DMSO) used to dilute the tested compounds were used as negative controls. Before the end of the incubation period (4 h for cell lines and 24 h for PBMCs), 20 µL of alamarBlue stock solution (resazurin, Sigma-Aldrich, St. Louis, MO, USA) was added to each well. Absorbance was measured at 570 nm (reduced) and 595 nm (oxidized) wavelengths, using a Spectramax 190 plate reader (Molecular Devices; Sunnyvale, CA, USA).

After determining the IC_50_ value for GANT61 (36 µM), all following assays were performed by using two concentrations: the IC_50_ value and half its concentration (18 µM). Then, 5-FU was used as a positive control in all further experimentation, due to its established clinical use as a chemotherapeutic protocol for patients with advanced OSCC.

### 4.4. Evaluation of the Expression of HH-Pathway-Related Proteins by Immunofluorescence

HSC3 cells were submitted to immunofluorescence to detect antibodies against proteins involved in the HH pathway (SHH, PTCH1, SMO, and GliLI1). In order to facilitate protein analysis, cells were grown without allowing cellular confluence on cell-specific coverslips (CellVIEW^TM^, Greiner Bio-One, Monroe, NC, USA) in the absence of fetal bovine serum. Coverslips were washed in sterile 1x PBS (pH 7.2) and fixed for 20 min in cold acetone/methanol. After washing with PBS, non-specific site blocking was performed though incubation in 1x PBS + 1% bovine serum albumin (BSA) solution for 30 min, followed by four additional washes. The following primary antibodies were diluted in 1x PBS + 1% BSA: mouse polyclonal against Sonic Hedgehog (1:500, Novus Biologicals, Clone 5 H4, Cat. number NBP2-22126, Novus Biologicals, Centennial, CO, USA), rabbit polyclonal against Gli1 (1:500, Novus Biologicals, Cat. number NB600-600, Novus Biologicals, Centennial, CO, USA), rabbit polyclonal against Patched 1 (1:500, Novus Biologicals, Cat. number NB200-118, Novus Biologicals, Centennial, CO, USA), and rabbit polyclonal against Smoothened (1:1000, Abcam, Cat. number AB72130, Abcam, Cambridge, MA, USA). Following primary antibody incubation overnight, cells were washed thrice with PBS and re-incubated under slow stirring for 1 h, at room temperature, with the following secondary antibodies diluted in PBS: Alexa Fluor 594 red-sheep anti-mouse IgG (1:1000, Molecular Probes, Eugene, OROregon, USA) and Alexa Fluor 488 green sheep anti-rabbit IgG (1:1000, Molecular Probes, Eugene, OROregon, USA). For all reactions, endogenous controls employing actin (1:10000, Invitrogen, Cat. number 15 G5 A11/E2, Invitrogen, Carlsbad, CA, USA) were performed following the same procedures described above, except for incubation with the primary antibody. Cell nuclei were then stained with 5 μg/mL of a fluorescent DNA labeling agent (4′,6-diamidino-2-phenylindol dihydrochloride, DAPI, Molecular Probes, Eugene, OR, USA) for 10 min, at room temperature. Cells were then washed with PBS, and morphological changes were evaluated and photographed under an inversion microscope (Leica DMi8, Wetzlar, Germany), using Leica Application Suite X software (XLAS X, Leica Microsystems, Wetzlar, Germany).

### 4.5. Western Blot Analysis of HH Pathway Component Expression

After culturing, HSC3 cells were washed with PBS and denatured by using cell lysis buffer containing 50 mM Tris-HCl (pH 7.4), 1% Triton X-100, 150 mM NaCl, 0.5 mM EGTA, 0.5 EDTA mM, and an anti-protease cocktail (Complete Pro-inhibitor Cocktail Tablets, Roche, Basel, Switzerland). Protein extracts (50 μg) were separated by SDS-PAGE and transferred to Hybond-C Extra (GE Healthcare, Chicago, IL, USA) nitrocellulose membranes. The following antibodies were used in the Western blot assay: mouse polyclonal against Sonic Hedgehog (1:500, Clone 5 H4, Cat. number NBP2-22126, Novus Biologicals, Centennial, CO, USA), rabbit polyclonal against Gli1 (1:500, Novus Biologicals, Cat. number NB600-600), rabbit polyclonal against Patched 1 (1:500, Novus Biologicals, Cat.number NB200-118), and rabbit polyclonal against Smoothened (1:1000, Cat. number AB72130, Abcam, Cambridge, MA, USA). The secondary antibodies were anti-mouse IgG (1:10,000, Santa Cruz Biotechnology, Santa Cruz, CA, USA) and anti-rabbit IgG (1:10,000, GE Healthcare, Chicago, IL, USA). Signal detection was performed by using an ECL + Chemiluminescence Detection System (PerkinElmer, Villebon sur-Yvette, France). The antibody actin (1:10000, Cat. Number15 G5 A11/E2, Invitrogen, Carlsbad, CA, USA) was selected as an endogenous control for analysis of HH pathway component expression in HSC3 cells.

The analysis of GANT61 effects on HH pathway protein expression (PTCH1, SHH, and GLI1) was performed by Western blotting, according to the protocol described above after 24 h of treatment. The polyclonal antibody Histone H3 (anti-rabbit, 1:2500, Abcam, Cat. number AB1791, Abcam, Cambridge, MA, USA) was selected as an endogenous control for post-treatment reactions.

### 4.6. HH Pathway Component Expression Following Inhibitor Treatment

#### 4.6.1. Total RNA Isolation

For total RNA isolation, HSC3 cells were plated on 6-well plates, at a density of 0.7 × 10^5^ cells/mL (5 mL/well volume), and incubated overnight, followed by treatment with the GLI inhibitor for 12 h at both concentrations (18 and 36 µM).

All experiments were carried out under DNAse/RNAse-free conditions. Silica microcolumns were used for RNA extraction, using an RNeasy Plus Mini Kit (QIAGEN, Hilden, Germany). Extracted RNA was analyzed for quantity and purity, using a NanoDrop^®®^ 1000 spectrophotometer (Thermo Fisher Scientific, Waltham, MA, USA). 

#### 4.6.2. Determination of HH Pathway Activity by Quantitative Polymerase Chain Reaction (qPCR)

Reverse transcription was performed by using Superscript VILO™ transcriptase enzyme (Invitrogen, Carlsbad, CA, USA). The cDNA samples were stored at −20 °C, until the time of use.

HH pathway component expression was evaluated in cells treated or not with GANT61 by qPCR, using TaqMan Gene Expression Assays^TM^ inventoried for the *PTCH1* (Hs00181117_m1), *GLI1* (Hs01110766_m1), *GLI2* (Hs01119974_m1), and *GLI3* (Hs00609233_m1) genes, as well as for B2 M (Hs99999907_m1). Reactions were performed a total volume of 20 μL in Fast 96-well sample blocks on an Applied Biosystems ViiA 7 system under standard cycling conditions for Fast-qPCR, as specified by the manufacturer (Applied Biosystems^TM^, Foster City, CA, USA).

Following amplification and dissociation runs, quantification cycle (Cq) values were obtained by using the ViiA7^TM^ System Operational Program (AppliedByosistems, Foster City, CA, USA). For relative quantification, the comparative method (2−∆∆Cq) described by Livak and Schmittgen, 2001 was used [47]. Cq values obtained for each sample were normalized by using the *B2M* reference gene and calibrated according to the Cq values obtained from the untreated HSC3 cell group (HSC3 NT). 

### 4.7. Evaluation of GANT61 Treatment on HSC3 Cell Viability, Cycle, and Pattern of Death

To study the effects of GANT61 on HSC3 cell viability, cycle, and death patterns, 4 mL of cellular solution (containing 0.7 × 10^5^ cells/mL) was placed on 6-well plates and incubated overnight, to allow adhesion. Next, cells were treated for 24, 48, or 72 h with GANT61 (18 or 36 μM, 50% or 100% of the determined IC_50_ value). For cell cycle analysis, the effects of GANT61 on HSC3 cells were also evaluated after 12 h of treatment. Negative controls were treated with the vehicle (0.2% DMSO) used to dilute the GLI inhibitor, and 5-fluorouracil (5-FU, 17 μM) was used as a positive control. All experiments were carried out in duplicate and repeated no less than three times. Cell viability was determined by the trypan blue exclusion method, in which 90 μL aliquots were collected at several incubation periods, stained with 10 μL trypan blue, and counted in a Neubauer chamber, under optical microscopy, at 20× magnification (Olympus CX41 Olympus, Tokyo, Japan).

### 4.8. Cell Cycle Determination and Internucleosomal DNA Fragmentation 

Cellular nuclear DNA content was evaluated by flow cytometry, to differentiate among cell cycle phases, using PI (propidium iodide) as a fluorogenic agent [48]. Following incubation with the GLI inhibitor, the supernatant was collected, and wells were washed with saline. Next, cells were trypsinized, then centrifuged (295× *g* for 5 min) together with the supernatant. Permeabilization was carried out by using a solution (300 µL) containing 0.1% triton X-100, 0.1% sodium citrate and 2 μg/mL of PI and 100 µg/mL RNase in distilled water, placed under dark conditions, at room temperature, for 30 min. Cells were then acquired (10,000 events/sample) and analyzed on a BD LSRFortessa flow cytometer, using BD FACSDiva software version 6.2 (Becton Dickinson Biosciences, San José, CA, USA). Proportions of fragmented internucleosomal DNA and cell cycle phases were determined by using Flowjo software v. 10 (Flowjo LCC, Ashland, OR, USA). Cells in sub-G1 phase were considered to present internucleosomal DNA fragmentation. Cellular debris was omitted from our analysis.

### 4.9. Determination of Phosphatidylserine Exteriorization

Following treatment with GANT61, cells were labeled with annexin V-FITC and PI to determine cell viability (classified as viable, early apoptosis, late apoptosis, and necrosis), according to the manufacturer’s protocol (Becton Dickinson Biosciences, San José, CA, USA). Cells were acquired (10,000 events/sample) and analyzed on a BD LSRFortessa flow cytometer, using BD FACSDiva software version 6.2 (Becton Dickinson Biosciences, San José, CA, USA). The proportion of cells undergoing apoptosis was determined by the percentage of annexin labeling as quantified by the FlowJo program, version 10 (Flowjo LCC, Ashland, OR, USA). Cellular debris was omitted from our analyses.

### 4.10. Morphological Analysis by Flow Cytometry

Morphological analysis of HSC3 cells was performed by flow cytometry, using FSC (size/volume) and SSC (granularity) parameters (FACSDiva). Cells were acquired (10,000 events/sample) and analyzed on a BD LSRFortessa flow cytometer, using the BD FACSDiva software version 6.2 (Becton Dickinson Biosciences, San José, CA, USA). Cellular debris was omitted from our analyses.

### 4.11. Statistical Analysis

Statistical analyses were performed, using GraphPad Prism v. 6.03 (GraphPad Software, Inc., San Diego, USA). Data were analyzed according to distribution along the normal Gaussian curve. IC_50_ values were obtained through non-linear regression, using results from three independent experiments carried out in duplicate. Differences between groups resulting from in vitro assays were evaluated by ANOVA (analysis of variance) testing, followed by the Student–Newman–Keuls test. This study considered a level of significance, corresponding to alpha (α) or “*p*” value, when results were less than or equal to 5%.

Regarding qPCR analysis, after performing amplification and dissociation runs, relative quantification (RQ) values were obtained for each sample, according to the ΔΔCQ comparative method and comparisons with the calibrator (untreated cells, RQ = 1.0), using the Gene Expression Suite™ program (Applied Biosystems, USA).

## Figures and Tables

**Table 1:** Cytotoxic activity of Doxorubicin, 5-FU, and the GANT61 in tumor and non-tumor cells.

Cells	Doxorubicin	5-FU	GANT61
**Tumor Cells**			
CAL27	3.09	30.8	>116
	2.22–4.3	13.6–70	
HSC3	0.34	16.75	35.9
	0.09–1.21	10.53–26.67	24.86–51.9
SCC4	0.07	N.d.	110.6
	0.05–0.09		77.46–157.89
SCC9	0.8	143.2	83.49
	0.25–2.31	82.02–250.1	27.3–255.1
SCC15	1.5	N.d.	72.74
	0.77–2.88		36.68–144.27
SCC25	1.4	N.d.	53.3
	0.61–2.92		34.53–82.19
HepG2	0.01	1.3	>116
	0.01–0.73	0.23–6.07	
HL60	0.01	12.6	>116
	0.01–0.05	9.53–16.68	
K562	0.05	18.14	>116
	0.04–0.09	9.76–33.51	
AGP01	1.89	N.d.	>116
	0.82–4.3		
APC02	6.8	N.d.	>116
	1.03–44.72		
APC03	2.3	N.d.	>116
	0.93–5.88		
HT-29	0.3	N.d.	>116
	0.26–0.44		
HCT-116	0.2	4.07	>116
	0.12–0.42	1.92–8.45	
**Cancer-Associated Fibroblasts**			
CAF 1	N.d.	>192	3.67
			0.48–27.65
CAF 2	N.d.	>192	71.46
			13.12–388.5
**Non-Tumor Cells**			
NOF	N.d.	39.59	>116
		12.3–123.4	
HaCat	0.1	N.d.	35.14
	0.01–0.55		18.27–67.59
PBMC	5.2	>192	87.54
	2.55–10.43		34.66–221

Data presented as IC_50_ values according to μM concentration and 95% confidence intervals obtained by non-linear regression of three independent experiments performed in duplicate (alamarBlue assay, 72 h of incubation). N.d. not determined. Note: IC_50_ values >116 (GANT61) and >192 (5-FU, Doxorubicin) indicate that the concentration established for each respective cell type was higher than the maximum value tested in the cytotoxicity assay.

## Supplementary Materials

Supplementary Materials can be found at https://www.mdpi.com/1422-0067/21/17/6076/s1. Figure S1: HH pathway components gene expression profiles in CAL27, HSC3 and SCC4 cells, Table S1: Tumor and non-tumor human cells used in cytotoxicity assay.

## References

[1] Bray F., Ferlay J., Soerjomataram I., Siegel R.L., Torre L.A., Jemal A. (2018). Global cancer statistics 2018: GLOBOCAN estimates of incidence and mortality worldwide for 36 cancers in 185 countries. CA Cancer J. Clin..

[2] El-naggar A., Chan J., Grandis J., Takata T., Slootweg P., World Health Organization (WHO) (2017). WHO Classification of Head and Neck Tumors.

[3] Chi A.C., Day T.A., Neville B.W. (2015). Oral cavity and oropharyngeal squamous cell carcinoma-an update. CA Cancer J. Clin..

[4] Johnson N.W., Jayasekara P., Amarasinghe A.A.H.K. (2011). Squamous cell carcinoma and precursor lesions of the oral cavity: Epidemiology and aetiology. Periodontol. 2000.

[5] Chung C.H., Dignam J.J., Hammond M.E., Klimowicz A.C., Petrillo S.K., Magliocco A., Jordan R., Trotti A., Spencer S., Cooper J.S. (2011). Glioma-associated oncogene family zinc finger 1 expression and metastasis in patients with head and neck squamous cell carcinoma treated with radiation therapy (RTOG 9003). J. Clin. Oncol..

[6] Dias R.B., De Araújo T.B.S., Freitas R., Rodrigues A.C.B.D.C., Sousa L.P., Sales C.B.S., Valverde L.D.F., Soares M.B.P., Dos Reis M.G., Della Coletta R. (2018). β-Lapachone and its iodine derivatives cause cell cycle arrest at G2/M phase and reactive oxygen species-mediated apoptosis in human oral squamous cell carcinoma cells. Free Radic. Boil. Med..

[7] Hitt R., Lopez-Pousa A., Martinez-Trufero J., Escrig V., Carles J., Rizo A., Isla M.D., Vega M.E., Marti J.L., Lobo F. (2005). Phase III study comparing cisplatin plus fluorouracil to paclitaxel, cisplatin, and fluorouracil induction chemotherapy followed by chemoradiotherapy in locally advanced head and neck cancer. J. Clin. Oncol..

[8] Buim M.E.C., Gurgel C.A.S., Ramos E.A.G., Lourenco S.V., Soares F.A. (2011). Activation of sonic hedgehog signaling in oral squamous cell carcinomas: A preliminary study. Hum. Pathol..

[9] Fan H.-X., Wang S., Zhao H., Liu N., Chen D., Sun M., Zheng J. (2014). Sonic hedgehog signaling may promote invasion and metastasis of oral squamous cell carcinoma by activating MMP-9 and E-cadherin expression. Med. Oncol..

[10] Agyeman A., Jha B.K., Mazumdar T., Houghton J.A. (2014). Mode and specificity of binding of the small molecule GANT61 to GLI determines inhibition of GLI-DNA binding. Oncotarget.

[11] Po A., Silvano M., Miele E., Capalbo C., Eramo A., Salvati V., Todaro M., Besharat Z.M., Catanzaro G., Cucchi D. (2017). Noncanonical GLI1 signaling promotes stemness features and in vivo growth in lung adenocarcinoma. Oncogene.

[12] Hardcastle Z., Mo R., Hui C.C., Sharpe P.T. (1998). The Shh signalling pathway in tooth development: Defects in Gli2 and Gli3 mutants. Developement.

[13] Burns M.A., Liao Z.W., Yamagata N., Pouliot G.P., Stevenson K.E., Neuberg D.S., Thorner A.R., Ducar M., Silverman E.A., Hunger S.P. (2018). Hedgehog pathway mutations drive oncogenic transformation in high-risk T-cell acute lymphoblastic leukemia. Leukemia.

[14] Deng H., Huang L., Liao Z., Liu M., Li Q., Xu R. (2020). Itraconazole inhibits the Hedgehog signaling pathway thereby inducing autophagy-mediated apoptosis of colon cancer cells. Cell Death Dis..

[15] Sharpe H.J., Pau G., Dijkgraaf G.J., Basset-Séguin N., Modrusan Z., Januario T., Tsui V., Durham A.B., Dlugosz A.A., Haverty P.M. (2015). Genomic analysis of smoothened inhibitor resistance in basal cell carcinoma. Cancer Cell.

[16] Kurebayashi J., Koike Y., Ohta Y., Saitoh W., Yamashita T., Kanomata N., Moriya T. (2017). Anti-cancer stem cell activity of a hedgehog inhibitor GANT61 in estrogen receptor-positive breast cancer cells. Cancer Sci..

[17] Desch P., Asslaber D., Kern D., Schnidar H., Mangelberger D., Alinger B., Stoecher M., Hofbauer S.W., Neureiter D., Tinhofer I. (2010). Inhibition of GLI, but not Smoothened, induces apoptosis in chronic lymphocytic leukemia cells. Oncogene.

[18] Lauth M., Bergström Å., Shimokawa T., Toftgård R. (2007). Inhibition of GLI-mediated transcription and tumor cell growth by small-molecule antagonists. Proc. Natl. Acad. Sci. USA.

[19] Wickström M., Dyberg C., Shimokawa T., Milosevic J., Baryawno N., Fuskevåg O.M., Larsson R., Kogner P., Zaphiropoulos P.G., Johnsen J.I. (2012). Targeting the hedgehog signal transduction pathway at the level of GLI inhibits neuroblastoma cell growth in vitro and in vivo. Int. J. Cancer.

[20] You M., Varona-Santos J., Singh S., Robbins D.J., Savaraj N., Nguyen D.M. (2014). Targeting of the Hedgehog signal transduction pathway suppresses survival of malignant pleural mesothelioma cells in vitro. J. Thorac. Cardiovasc. Surg..

[21] Yan M., Wang L., Zuo H., Zhang Z., Chen W., Mao L., Zhang P. (2011). HH/GLI signalling as a new therapeutic target for patients with oral squamous cell carcinoma. Oral Oncol..

[22] Momose F., Araida T., Negishi A., Ichijo H., Shioda S., Sasaki S. (1989). Variant sublines with different metastatic potentials selected in nude mice from human oral squamous cell carcinomas. J. Oral Pathol. Med..

[23] Erdem N.F., Carlson E.R., Gerard D.A., Ichiki A.T. (2007). Characterization of 3 oral squamous cell carcinoma cell lines with different invasion and/or metastatic potentials. J. Oral Maxillofac. Surg..

[24] Gioanni J., Fischel J.-L., Lambert J.-C., Demard F., Mazeau C., Zanghellini E., Ettore F., Formento P., Chauvel P., Lalanne C.-M. (1988). Two new human tumor cell lines derived from squamous cell carcinomas of the tongue: Establishment, characterization and response to cytotoxic treatment. Eur. J. Cancer Clin. Oncol..

[25] Jiang L., Ji N., Zhou Y., Li J., Liu X., Wang Z., Chen Q., Zeng X. (2009). CAL 27 is an oral adenosquamous carcinoma cell line. Oral Oncol..

[26] Rheinwald J.G.A., Beckett M. (1981). Tumorigenic keratinocyte lines requiring anchorage and fibroblast support cultured from human squamous cell carcinomas. Cancer Res..

[27] Samarzija I., Beard P. (2012). Hedgehog pathway regulators influence cervical cancer cell proliferation, survival and migration. Biochem. Biophys. Res. Commun..

[28] Fu J., Rodova M., Roy S.K., Sharma J., Singh K.P., Srivastava R., Shankar S. (2012). GANT-61 inhibits pancreatic cancer stem cell growth in vitro and in NOD/SCID/IL2R gamma null mice xenograft. Cancer Lett..

[29] Huang L., Walter V., Hayes D.N., Onaitis M. (2014). Hedgehog-GLI signaling inhibition suppresses tumor growth in squamous lung cancer. Clin. Cancer Res..

[30] Kiesslich T., Mayr C., Wachter J., Bach D., Fuereder J., Wagner A., Alinger B., Pichler M., Di Fazio P., Ocker M. (2014). Activated hedgehog pathway is a potential target for pharmacological intervention in biliary tract cancer. Mol. Cell. Biochem..

[31] Pan D., Li Y., Li Z., Wang Y., Wang P., Liang Y. (2012). Gli inhibitor GANT61 causes apoptosis in myeloid leukemia cells and acts in synergy with rapamycin. Leuk. Res..

[32] Srivastava R.K., Kaylani S.Z., Edrees N., Li C., Talwelkar S.S., Xu J., Palle K., Pressey J.G., Athar M. (2014). GLI inhibitor GANT-61 diminishes embryonal and alveolar rhabdomyosarcoma growth by inhibiting Shh/AKT-mTOR axis. Oncotarget.

[33] Pignon J.-P., Syz N., Posner M., Olivares R., Le Lann L., Yver A., Dunant A., Lewin F., Dalley D.N., Paccagnella A. (2004). Adjusting for patient selection suggests the addition of docetaxel to 5-fluorouracil–cisplatin induction therapy may offer survival benefit in squamous cell cancer of the head and neck. Anti-Cancer Drugs.

[34] Cohen R.B. (2014). Current challenges and clinical investigations of epidermal growth factor receptor (EGFR)- and ErbB family-targeted agents in the treatment of head and neck squamous cell carcinoma (HNSCC). Cancer Treat. Rev..

[35] Marigo V., Johnson R.L., Vortkamp A., Tabin C.J. (1996). Sonic hedgehog differentially regulates expression of GLI and GLI3 during limb development. Dev. Boil..

[36] Thompson M.C., Fuller C., Hogg T.L., Dalton J.D., Finkelstein D., Lau C.C., Chintagumpala M., Adesina A., Ashley D.M., Kellie S.J. (2006). Genomics identifies medulloblastoma subgroups that are enriched for specific genetic alterations. J. Clin. Oncol..

[37] Rudin C.M., Hann C.L., Laterra J., Yauch R.L., Callahan C.A., Fu L., Holcomb T., Stinson J., Gould S.E., Coleman B. (2009). Treatment of medulloblastoma with hedgehog pathway inhibitor GDC-0449. N. Engl. J. Med..

[38] Beachy P.A., Hymowitz S.G., Lazarus R.A., Leahy D.J., Siebold C. (2010). Interactions between Hedgehog proteins and their binding partners come into view. Genes Dev..

[39] Haaf A.T., Bektas N., Von Serényi S., Losen I., Arweiler E.C., Hartmann A., Knüchel R., Dahl E. (2009). Expression of the glioma-associated oncogene homolog (GLI) 1 in human breast cancer is associated with unfavourable overall survival. BMC Cancer.

[40] Blotta S., Jakubikova J., Calimeri T., Roccaro A.M., Amodio N., Azab A.K., Foresta U., Mitsiades C.S., Rossi M., Todoerti K. (2012). Canonical and noncanonical Hedgehog pathway in the pathogenesis of multiple myeloma. Blood.

[41] Lim C.B., Prele C.M., Baltic S., Arthur P.G., Creaney J., Watkins D.N., Thompson P.J., Mutsaers S.E. (2015). Mitochondria-derived reactive oxygen species drive GANT61-induced mesothelioma cell apoptosis. Oncotarget.

[42] Marigo V., Tabin C.J. (1996). Regulation of patched by sonic hedgehog in the developing neural tube. Proc. Natl. Acad. Sci. USA.

[43] Benvenuto M., Masuelli L., De Smaele E., Fantini M., Mattera R., Cucchi D., Bonanno E., Di Stefano E., Frajese G.V., Orlandi A. (2016). In vitro and in vivo inhibition of breast cancer cell growth by targeting the Hedgehog/GLI pathway with SMO (GDC-0449) or GLI (GANT-61) inhibitors. Oncotarget.

[44] Lin Z., Li S., Sheng H., Cai M., Ma L.Y.S., Hu L., Xu S., Yu L.S., Zhang N. (2016). Suppression of GLI sensitizes medulloblastoma cells to mitochondria-mediated apoptosis. J. Cancer Res. Clin. Oncol..

[45] Sobral L.M., Bufalino A., Lopes M.A., Graner E., Salo T., Coletta R.D. (2011). Myofibroblasts in the stroma of oral cancer promote tumorigenesis via secretion of activin A. Oral Oncol..

[46] Ahmed S.A., Gogal R.M., Walsh J.E. (1994). A new rapid and simple non-radioactive assay to monitor and determine the proliferation of lymphocytes: An alternative to [3H]thymidine incorporation assay. J. Immunol. Methods.

[47] Livak K.J., Schmittgen T.D. (2001). Analysis of relative gene expression data using real-time quantitative PCR and the 2(-Delta Delta C(T)) method. Methods.

[48] Nicoletti I., Migliorati G., Pagliacci M., Grignani F., Riccardi C. (1991). A rapid and simple method for measuring thymocyte apoptosis by propidium iodide staining and flow cytometry. J. Immunol. Methods.

