# Supplementary material for: GANT61 Reduces Hedgehog Molecule (GLI1) Expression and Promotes Apoptosis in Metastatic Oral Squamous Cell Carcinoma Cells
Source: Int J Mol Sci. 2020 Aug 24;21(17):6076. doi: 10.3390/ijms21176076 (PMC7503713; doi:10.3390/ijms21176076)
Supplement: Supplementary file 1 [file ijms-21-06076-s001.pdf]

International Journal of
Molecular Sciences

Supplementary Material

GANT61 REDUCES HEDGEHOG MOLECULE EXPRESSION AND PROMOTES APOPTOSIS IN ORAL SQUAMOUS CELL CARCINOMA

Taís Bacelar Sacramento de Araújo[1,2], Leonardo de Oliveira Siquara da Rocha[1,2], Manuela Torres Andion Vidal[1,2], Paulo Lucas Cerqueira Coelho[1], Mitermayer Galvão dos Reis[1,2], Bruno Solano de Freitas Souza[1,3], Milena Botelho Pereira Soares[1], Ricardo Della Coletta[4], Daniel Pereira Bezerra[1], Rosane Borges Dias[1,2], Clarissa Araújo Gurgel Rocha[1,2]

TABLES

Table S1. Tumor and non-tumor human cells used in cytotoxicity assay.

Cell line	Histological type	Source
CAL 27	Oral squamous cell carcinoma	Antoine Lacassagne Center Bio-Oncology Lab Nice – France
HSC3	Oral squamous cell carcinoma	Health Sciences Research Resource Bank in Japan
SCC4, SCC9, SCC15 e SCC25	Oral squamous cell carcinoma	Sidney Farber Cancer Institute, Cell Regulation and Growth Division and Physiology Department, Harvard Medical School
HepG2	Hepatocellular carcinoma	Wistar Anatomy and Biology Institute, Philadelphia, Pennsylvania
HL-60	Acute promyelocytic leukemia	Mercy Pediatric Hospital – USA
K562	Chronic myelogenic leukemia	Splenic Physiopathology and Cytogenetics Laboratories, University of Tennessee Hospital and Research Center
AGP01	Peritoneal gastric adenocarcinoma	Federal University of Pará - Brazil
ACP02	Gastric adenocarcinoma	Federal University of Pará - Brazil
ACP03	Gastric adenocarcinoma	Federal University of Pará - Brazil
HT-29	Colon adenocarcinoma	Sloan Kettering Institute – USA
HCT-116	Colon carcinoma	Biochemistry Department of the University of Alabama – USA

CAF1	Oral cancer-associated fibroblast (primary cell)	Dentistry School of the Federal University of Campinas – UNICAMP/Piracicaba
CAF2	Oral cancer-associated fibroblast (primary cell)	Dentistry School of the Federal University of Campinas – UNICAMP/Piracicaba
NOF	Normal oral fibroblast (primary cell)	Dentistry School of the Federal University of Campinas – UNICAMP/Piracicaba
HaCaT	Non-transformed immortalized normal keratinocytes	*In vitro* Carcinogenesis and Differentiation Division, Biochemistry Institute, Cancer Research Center, Germany

FIGURES

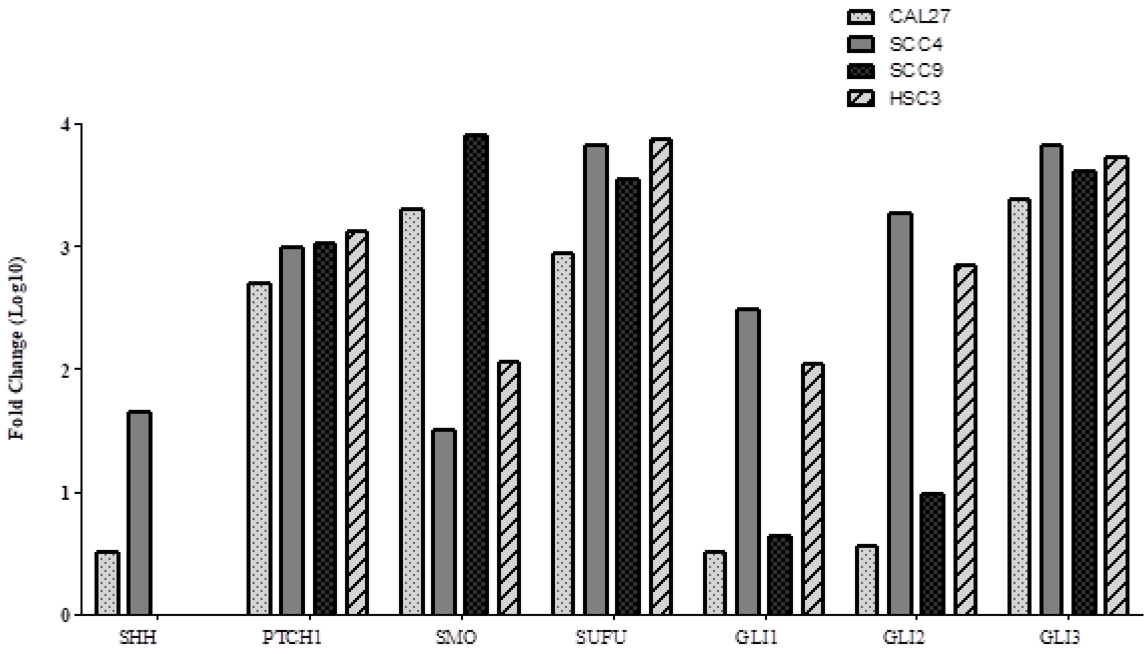

Figure S1. HH pathway components gene expression profiles in CAL27, HSC3 and SCC4 cells.
